# Supplementary material for: Epidemiological Features of Human Norovirus Genotypes before and after COVID-19 Countermeasures in Osaka, Japan
Source: Viruses. 2024 Apr 22;16(4):654. doi: 10.3390/v16040654 (PMC11055107; doi:10.3390/v16040654)
Supplement: Supplementary file 1 [file viruses-16-00654-s001.zip › viruses-2959844-supplementary.pdf]

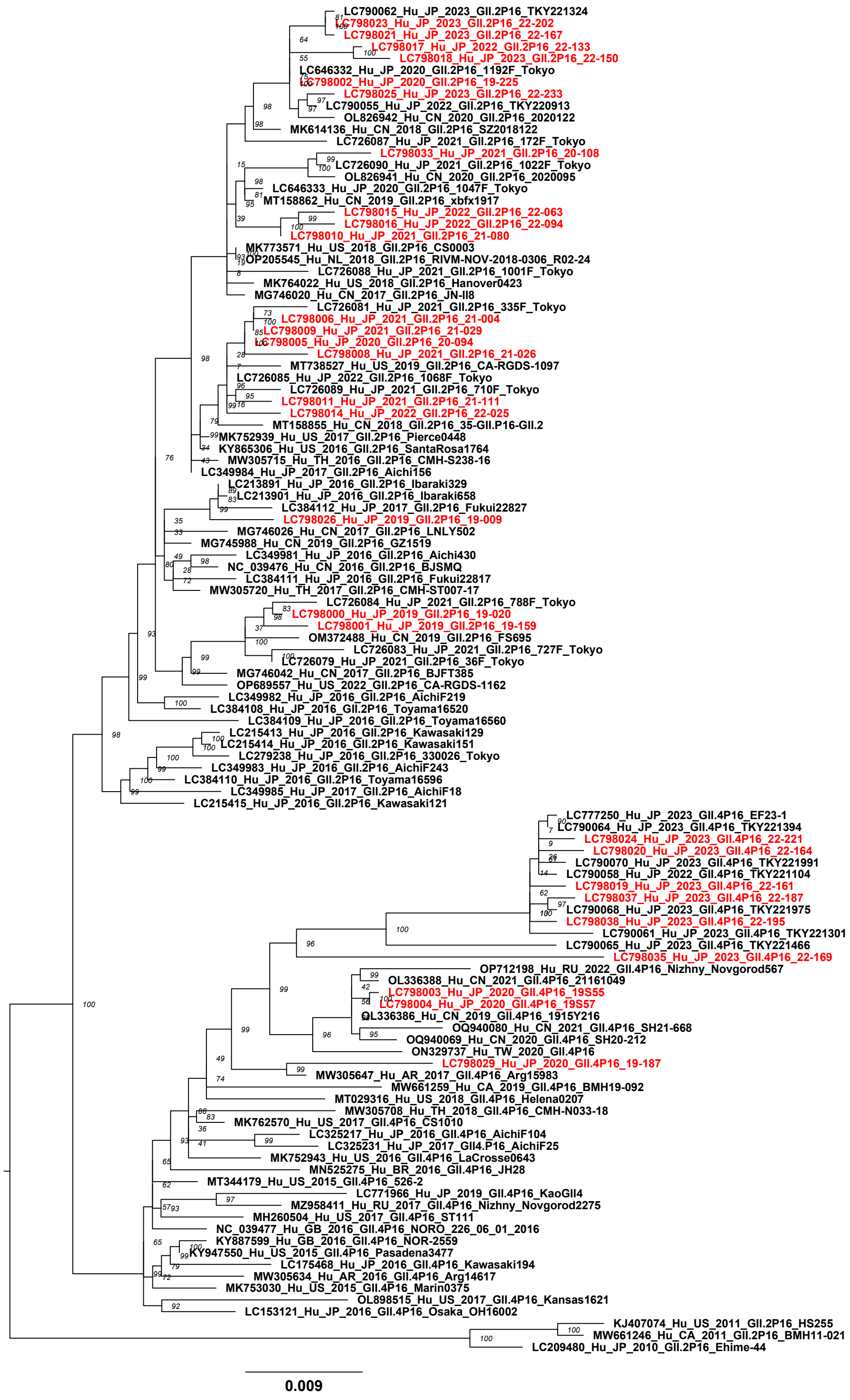

Figure S1. Maximum-likelihood tree of HuNoV P-type GII.P16 based on RdRp (1533 bp). One hundred and fourteen RdRp sequences including 28 Osaka strains obtained during the present study period and 86 global strains are labeled in red and black, respectively. Seven out of the 28 Osaka strains, LC798017, LC798021, LC798023, LC798025, LC798035, LC798037, and LC798038, were collected from the outside of the areas covered by the surveillance systems and were also included as reference strains. Numbers on branches indicate bootstrap support values.

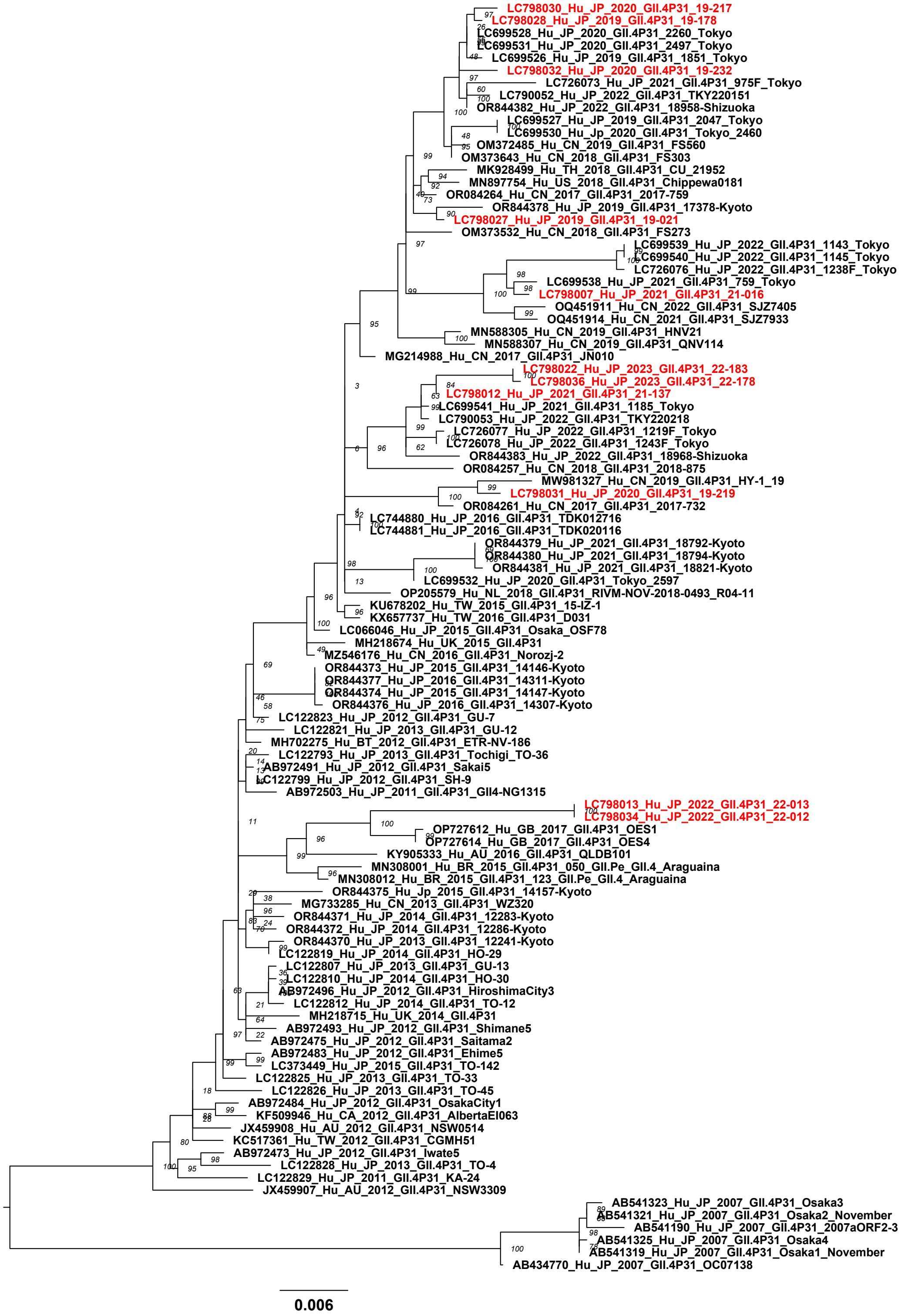

Figure S2. Maximum-likelihood tree of HuNoV P-type GII.P31 based on RdRp (1533 bp). One hundred and two RdRp sequences including 11 Osaka strains obtained during the present study period and 91 global strains are labeled in red and black, respectively. Numbers on branches indicate bootstrap support values.

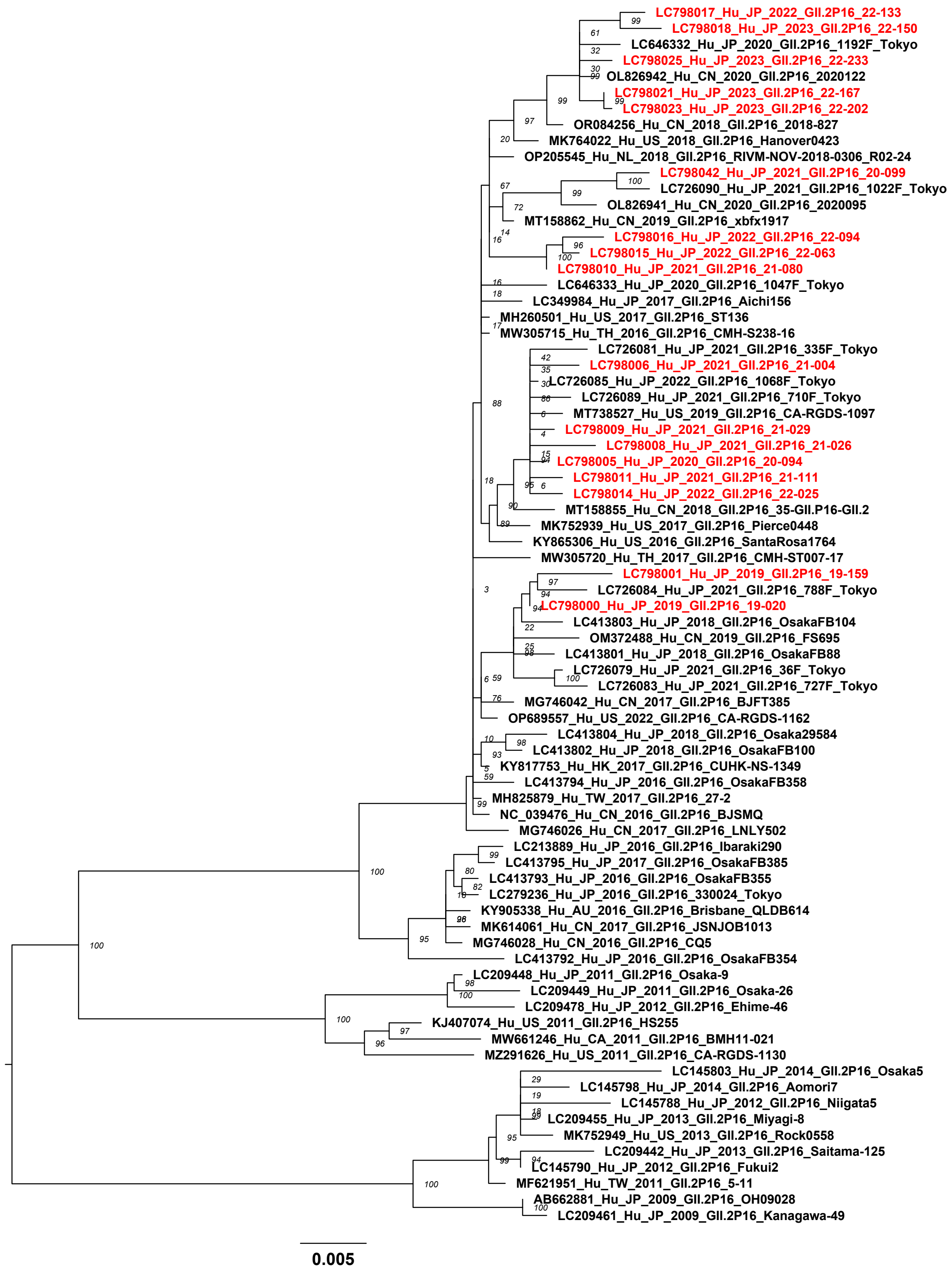

Figure S3. Maximum-likelihood tree of HuNoV genotype GII.2 based on VP1 (1629 bp). Seventy six VP1 sequences including 17 Osaka strains obtained during the present study period and 59 global strains are labeled in red and black, respectively. Four out of 17 Osaka strains, LC798017, LC798021, LC798023, and LC798025, were collected from the outside the areas covered by the surveillance systems and were also included as reference strains. Numbers on branches indicate bootstrap support values.

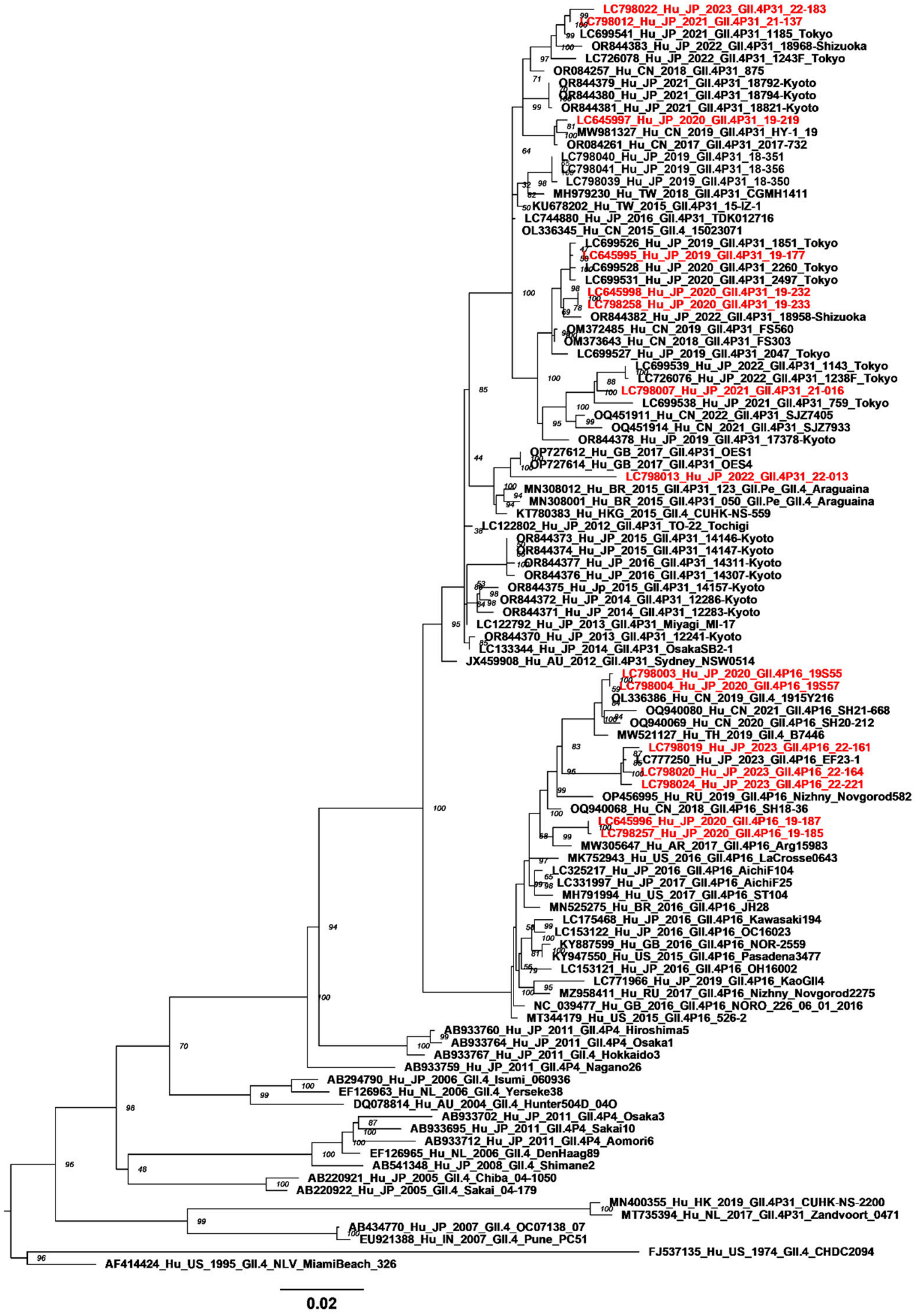

Figure S4. Maximum-likelihood tree of HuNoV genotype GII.4 based on VP1 (1623 bp). One hundred and three VP1 sequences including 15 Osaka strains obtained during the present study period and 88 global strains are labeled in red and black, respectively. Four out of 15 Osaka strains, LC645995–LC645998, had been reported in the previous study [40], and were also included. Three Osaka strains, LC798039–LC798041, were collected before the present study period and were also included as reference strains. Numbers on branches indicate bootstrap support values.
